# *STAG3* truncating variant as the cause of primary ovarian insufficiency

**DOI:** 10.1038/ejhg.2015.107

**Published:** 2015-06-10

**Authors:** Polona Le Quesne Stabej, Hywel J Williams, Chela James, Mehmet Tekman, Horia C Stanescu, Robert Kleta, Louise Ocaka, Francesco Lescai, Helen L Storr, Maria Bitner-Glindzicz, Chiara Bacchelli, Gerard S Conway, GOSgene

**Affiliations:** 1 Department of Genetics and Genomic Medicine, UCL Institute of Child Health, London, UK; 2 Division of Medicine, UCL, London, UK; 3 Centre for Endocrinology, William Harvey Research Institute, Barts and the London School of Medicine and Dentistry, Queen Mary University of London, London, UK; 4 Reproductive Medicine Unit, Institute for Women's Health, University College London Hospitals, London, UK

## Abstract

Primary ovarian insufficiency (POI) is a distressing cause of infertility in young women. POI is heterogeneous with only a few causative genes having been discovered so far. Our objective was to determine the genetic cause of POI in a consanguineous Lebanese family with two affected sisters presenting with primary amenorrhoea and an absence of any pubertal development. Multipoint parametric linkage analysis was performed. Whole-exome sequencing was done on the proband. Linkage analysis identified a locus on chromosome 7 where exome sequencing successfully identified a homozygous two base pair duplication (c.1947_48dupCT), leading to a truncated protein p.(Y650Sfs*22) in the *STAG3* gene, confirming it as the cause of POI in this family. Exome sequencing combined with linkage analyses offers a powerful tool to efficiently find novel genetic causes of rare, heterogeneous disorders, even in small single families. This is only the second report of a *STAG3* variant; the first *STAG3* variant was recently described in a phenotypically similar family with extreme POI. Identification of an additional family highlights the importance of *STAG3* in POI pathogenesis and suggests it should be evaluated in families affected with POI.

## Introduction

Primary ovarian insufficiency (POI) is defined as a post pubertal hypergonadotropic hypogonadism in women under the age of 40 years. POI presenting with secondary amenorrhoea affects an estimated 1:100 women.^1^ However, early onset forms of POI, presenting as primary amenorrhoea with lack of any pubertal development, affect an estimated 1:100 000 females. Women with POI report high levels of depression and low levels of self-esteem with negative effects on sexuality.^2^ The cause of POI is not known in the majority of cases.

A growing number of genes have been identified, which harbour variants that affect function associated with POI. The strength of evidence linking each anomaly with POI is variable. For example, the association of the fragile site mental retardation 1 gene (*FMR1*) with POI is widely reported, whereas in others only a single case represents the link (*STAG3, NOGGIN*).^3, 4, 5^ In some cases, the genetic link may be indirect, such as galactose-1-phosphate uridyltransferase (*GALT*) variants causing biochemical damage of the ovary and autoimmune regulator (*AIRE*) variants, which trigger autoimmune damage.^6^ Other possible genetic causes of POI are variants in *FMR2*, *NR5A1*, *FOXL2*, *INHAGDF9*, *NOBOX* and *PGRMC1*, *BMP15* genes on the X chromosome (for recent review see Fortuño and Labarta^7^). Taken altogether, known genes causing ovarian insufficiency are known to account for less than 5% of cases; however, family studies suggest that 20–30% of women with POI have an affected relative.^8^ As mentioned above, it has recently been reported that POI can result from truncating variant in stromal antigene 3 gene (*STAG3*), which encodes a subunit of cohesin; a large ring-shaped protein complex essential for proper pairing and segregation of chromosomes during meiosis, which is required for gametogenesis and fertility.^5^ In mammals, there are four known meiosis-specific cohesin core subunits: SMC1β, RAD21L, REC8 and STAG3. Stag3-deficient female mice showed a lack of oocytes and ovarian follicles indicating a severe ovarian dysgenesis.^9^ Similarly, Rec8 null mice of both sexes have germ cell failure and are sterile and Rad21l-deficient females are fertile but develop an age-dependent sterility.^10, 11^

In this report, applying a combined linkage analysis and whole-exome sequencing approach, we replicate the role of *STAG3* as the cause of POI by identifying a novel truncating variant in a consanguineous Lebanese family.

## Patients and methods

### Patients

Two sisters with POI presented with primary amenorrhoea and complete lack of any pubertal development. The proband presented at age 11 years with the absence of pubertal development in contrast to her unaffected sister who had demonstrated signs of puberty from age 9 years, and who subsequently progressed through normal puberty. The proband commenced oestrogen treatment at 13 years, with menarche achieved at 15 years of age. Breast development remained incomplete and she underwent breast augmentation at age 17 years. Investigations at presentation included LH 31iu/l, FSH 136 IU/l, oestradiol undetectable <37 pmol/l and inhibin B undetectable <15.6 pg/ml. Ovarian autoantibodies negative, karyotype 46,XX. Trans-abdominal ultrasound performed at age 19 years confirmed the presence of a normal uterus with bilateral 19 mm adnexal structures consistent with streak gonads.

The proband's younger sister, aged 16 years, also presented at 11 years of age and was started on oestrogen with satisfactory development of secondary sex characteristics. Investigations at presentation included LH 62iu/l, FSH 130 IU/l, oestradiol undetectable <37 pmol/l and inhibin B undetectable <15.6 pg/ml. Ovarian autoantibodies negative, karyotype 46,XX. Menarche occurred at age 15 years. Trans-abdominal ultrasound at the age of 16 years confirmed the presence of a small but morphologically normal uterus with adnexal structures consistent with streak gonads. Height was within the normal range for both women.

**Figure 1:**
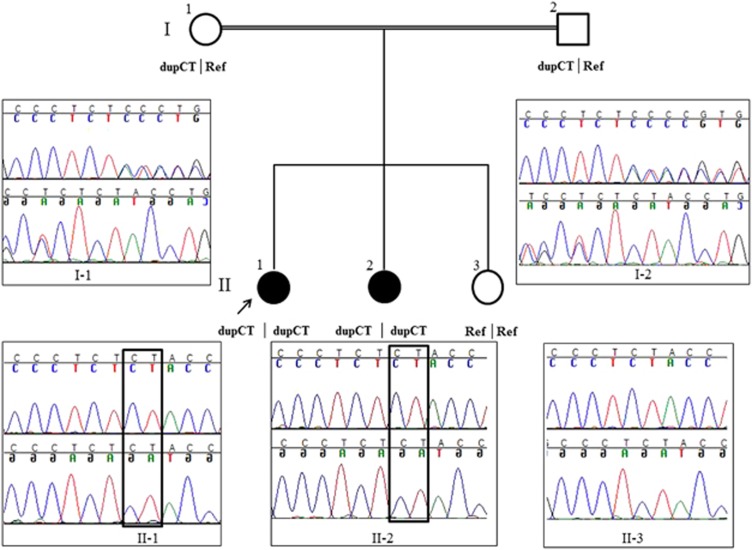
Segregation of *STAG3* c.1947_48dupCT resulting in STAG3 p.(Y650Sfs*22) in a Lebanese consanguineous family. Electropherograms generated by Sanger sequencing. Proband (II-1) is indicated by an arrow. Unaffected sister (II-3) is homozygous for the reference sequence (Ref), affected sisters (II-1 and II-2) are *STAG3* c.1947_48dupCT homozygous (CT duplication is depicted in a box) and the parents are heterozygous.

The subjects were members of a consanguineous Lebanese family and their parents were first cousins (Figure 1). No other females in the family were affected. No other clinical features of note were reported. Informed consent to the study was obtained from all participants and genetic studies were approved by the joint UCL/UCLH ethics committee. Genomic DNA of the proband and family members was extracted from peripheral blood by standard methods.

### Linkage analysis

Multipoint parametric linkage studies of five members of the family, including the two affected individuals and an unaffected sister, was performed using the HumanCytoSNP-12v2-1_H Beadarray (Illumina, Inc, San Diego, CA, USA) as per the manufacturer's instructions as published previously.^12^ A recessive transmission model with complete penetrance and a disease allele frequency of 0.0001 was used for linkage analysis.

### Whole-exome sequencing

Whole-exome capture was performed with Agilent SureSelect version 3 (Agilent, Santa Clara, CA, USA), according to the manufacturer's protocol on the proband (II-1 in Figure 1). Enriched libraries were sequenced on the Illumina HiSeq2000. Sequencing reads passing quality filters were aligned to the reference genome build GRGh37/hg19 using Burrows-Wheeler Aligner (BWA) algorithm and for variant calling we applied GATK base quality score recalibration, indel realignment, duplicate removal and performed SNP and INDEL discovery and genotyping using standard hard filtering parameters or variant quality score.^13, 14^

The variant annotation and interpretation analyses were generated through the use of Ingenuity Variant Analysis software version 3.0.20140422 (http://www.ingenuity.com/variants) from Ingenuity Systems. Exonic and cryptic splice site variants, which were homozygous in proband, resided in the linkage region, had a call quality ≥20, read depth ≥10 and were not present in the internal database of 358 exomes or observed with allele frequency ≥0.5% in public exome databases (1000 genomes, NHLBI ESP exomes) were kept.

### Sanger sequencing

The *STAG3* variant c.1947_48dupCT (chr7.hg19:g.99798478_79dupCT) identified by exome sequencing was validated by Sanger sequencing. Segregation was confirmed in all family members (Figure 1). The following primers were designed using Primer3 online software and used to amplify 432 nucleotides long fragment surrounding the site of c.1947_48dupCT in *STAG3*: forward: 5′-GCTTGGAGAAGGTAGGGAGA-3′ and reverse: 5′-CAGCTCTTCAAGCTCCTGC-3′.^15^ The variant was submitted to Leiden Open Variation Database (http://www.lovd.nl/STAG3; patient IDs: 0029771 and 00029822).^16^

## Results

### Linkage analysis

**Figure 2:**
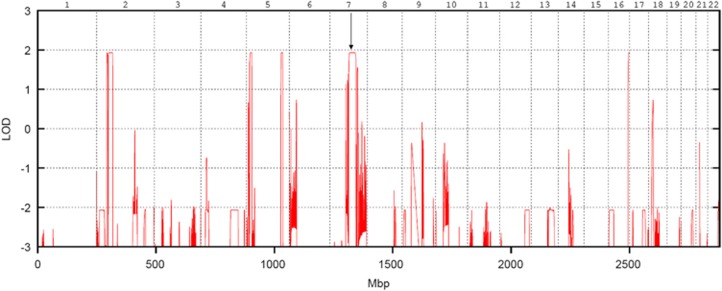
Linkage analysis of a Lebanese consanguineous family presenting with primary ovarian insufficiency. Linkage plot showing logarithm of odds (LOD) scores across the whole genome. Arrow indicates linkage to *STAG3*.

To narrow down the region of interest, we performed multipoint parametric linkage analysis, which showed five linked regions on chromosomes 2, 5, 7 and 16, respectively, with a logarithm of odds (LOD) score for this consanguineous pedigree matching the one expected of 1.93 (Figure 2). Other regions within the genome could be excluded being causative for POI by this analysis (LOD score <−2.0).

### Exome sequencing

We performed whole-exome sequencing on DNA from the proband (individual II-1; Figure 1). 66.6 Gb of sequence was generated to achieve a mean 48x coverage. Four variants passed upstream filtering: three missense variants were present in the Single-Nucleotide Polymorphism Database at frequencies below 0.1% these were rs148227466, rs150823294 and rs139520739. The remaining variant was a two base pair duplication in exon 19 (exons numbered according to Ensembl transcript ENST00000426455) of the *STAG3* gene (chr7.hg19:g.99798478_79dupCT; c.1947_48dupCT in NM_012447.3) causing a frame-shift p.(Y650Sfs*22) (NP_036579). A one base pair homozygous deletion inducing a frame-shift variant p.(F187fs*7) in *STAG3* has recently been reported as the cause of premature ovarian failure in a large consanguineous Palestinian family.^5^ STAG3 p.(Y650Sfs*22) was therefore the best disease-causing candidate variant. *STAG3* lies in a region that was linked to POI with an expected LOD score of 1.93 (Figure 2).

### Sanger sequencing

The *STAG3* variant was confirmed by Sanger sequencing and segregated with the POI phenotype (Figure 1).

## Discussion

Exome sequencing has transformed the identification of rare Mendelian disease genes as it searches for the causative variants in an unbiased manner.^17^ In our study, we identified the POI affecting variant in *STAG3* in a Lebanese consanguineous family with two affected and one unaffected females using a combination of exome sequencing in the proband and linkage analysis. Direct whole-exome sequencing of proband only results in 531 rare, homozygous variants; 479 (90.2%) of these variants reside outside linkage regions and hence do not segregate with the POI phenotype. Linkage analysis was therefore crucial in positioning of the phenotype affecting variant.

By sifting 54 667 variants identified in proband's exome through a series of carefully designed filters, we were able to narrow the search for disease-causing variants to four candidate variants. The homozygous TC duplication (c.1947_48dupCT), resulting in a truncated STAG3 protein p.(Y650Sfs*22), was the most likely function affecting variant because of linkage to the trait, the severity of the variant and the involvement of *STAG3* in meiosis, which is vital for early fetal ovary development.^18^ *STAG3* has 32 exons, its transcript is 4450 nucleotides and the protein is 1225 amino acids long. The c.1947_48dupCT results in a frame-shift at amino-acid position 650 followed by a premature stop codon with exons 19–32 being omitted. The transcript would either result in a truncated protein or would undergo nonsense-mediated decay resulting in a loss of function.

The confirmation of STAG3 p.(Y650Sfs*22) as variant affecting POI came when, during the preparation of this manuscript, Caburet *et al.* published a different *STAG3* truncating variant(p.F187fs*7) as the cause of POI in a Palestinian family.^5^ Caburet *et al.* confirmed that STAG3 loss of function is associated with POI by generating a mouse model carrying an insertional null variant in *Stag3,* resulting in sterile mice with fetal oocytes arrested at early prophase I, leading to oocyte depletion at 1 week of age.^5^ It is always difficult to assign causality to a gene from the evidence of a single family even with strong evidence from animal models and so our replication of the findings of Caburet *et al.*^5^ elevate *STAG3* from the level of a possible POI-causative gene to a confirmed risk gene that has now been identified in two unrelated families.

An interesting observation in the study by Caburet and colleagues was that the male Stag3^−/−^ mice were also infertile.^5^ In our study, the proband had no brothers, and so the effects, if any, in males cannot be confirmed.

STAG3 is part of the cohesin complex that is conserved from yeast to man and has the ability to hold together two DNA segments within its ring-shaped structure. When the two segments belong to sister chromatids, cohesin is mediating cohesion, which is essential for chromosome segregation in mitosis and meiosis and for homologous DNA repair. Variants in cohesin and its interacting factors have been associated with conditions as diverse as cancer and developmental syndromes known as ‘cohesinopathies'.^19^ This is particularly interesting given the previous report of a gonadoblastoma on the right ovary and a complex tumour in the left ovary at the age of 19 years,^5^ a finding not shared by the affected patients in our study who were last examined at the age of 16 and 19 years, respectively. The risk of gonadal tumour and implications with regard to genetic counselling therefore remains to be clarified.

The clinical findings of the proband and her affected sister in our study showed a severe phenotype with streak gonads, similar to the phenotype of the affected sisters reported previously;^5^ it may therefore be a good candidate gene for other POI cases with severe phenotypes. However, linkage analysis and homozygosity mapping in three additional POI-affected consanguineous families excluded *STAG3* as a possible candidate gene (unpublished data), a finding in keeping with Caburet *et al.* who also ruled out *STAG3* locus in four other families with obvious recessive inheritance.

Discovery of variants causing POI is challenging due to heterogeneity, limitation of informative families due to infertility and the small number of known POI-causing genes. Identification of two distinctive homozygous truncating variants in *STAG3* in consanguineous families of Lebanese (our study) and Palestinian origin put *STAG3* firmly on the list of candidate genes for POI, especially in patients presenting with primary amenorrhoea. It also implicates potential involvement of other meiosis-specific genes of the cohesin complex in POI pathogenesis.^5^
